# Predicting Anxiety in Secondary School Teachers: A Transdiagnostic Approach

**DOI:** 10.3390/ejihpe16080112

**Published:** 2026-07-31

**Authors:** Paula Rodríguez-Fernández, Ema Di Petrillo, Matteo Villanova, Valeria Caggiano, Juana María Gutiérrez-Caballero, Luna Blázquez-Gutiérrez, Alberto Blázquez-Manzano, Jerónimo Javier Gonález-Bernal

**Affiliations:** 1 Department of Education and Educational Innovation, Faculty of Law, Education and Humanities, Universidad Europea de Madrid, 28670 Madrid, Spain; 2 Department of Health Sciences, University of Burgos, 09001 Burgos, Spain; dipetrillo@romebusinessschool.com (E.D.P.); jejavier@ubu.es (J.J.G.-B.); 3 Department of Education, Rome Business School, 00196 Rome, Italy; 4 Department of Education, Roma Tre University, 00154 Rome, Italy; matteo.villanova@uniroma3.it (M.V.); valeria.caggiano@uniroma3.it (V.C.); 5 Department of Psychology and Anthropology, University of Extremadura, 06006 Badajoz, Spain; jugutierrezc@unex.es; 6 Department of Philosophy, Logic and Aesthetics, University of Salamanca, 37007 Salamanca, Spain; id00931792@usal.es; 7 Department of Sports, Regional Government of Extremadura, 06800 Mérida, Spain; alberto.blazquez@juntaex.es

**Keywords:** multidimensional anxiety, physical anxiety, cognitive anxiety, social anxiety, teachers, transdiagnostic approach, intolerance of uncertainty, fear of negative evaluation, procrastination, secondary school teachers

## Abstract

(1) Background: Teacher mental health is a growing concern due to the high emotional and professional demands of teaching. Anxiety is particularly prevalent among teachers, yet it is often conceptualized as a unitary construct, overlooking its multidimensional nature. This study examined the role of transdiagnostic psychological variables and sociodemographic factors as predictors of different dimensions of anxiety (physical, cognitive, and social) in secondary school teachers. (2) Methods: A total of 444 Spanish secondary school teachers (Mage = 41.76, SD = 10.80; 50% women) participated. Data were collected via an online questionnaire. A quantitative, non-experimental, cross-sectional design was used. Pearson correlations and hierarchical multiple regression analyses were conducted to analyze associations and predictive relationships. (3) Results: Anxiety showed a multidimensional structure, with hierarchical regression models explaining a substantial proportion of variance (R^2^ = 0.26–0.47). Transdiagnostic psychological variables accounted for the largest increases in explained variance beyond sociodemographic and work-related factors. Intolerance of uncertainty was the most consistent predictor across all anxiety dimensions, whereas fear of negative evaluation was the strongest predictor of social anxiety and was also associated with physical and cognitive anxiety. Procrastination predicted cognitive and social anxiety. Female teachers reported higher anxiety levels, while teaching experience showed a protective effect on cognitive anxiety. (4) Conclusions: Findings support a transdiagnostic framework and highlight the central role of psychological processes, particularly intolerance of uncertainty and fear of negative evaluation, in explaining teacher anxiety. Interventions targeting these shared mechanisms may contribute to improving teachers’ mental health and wellbeing.

## 1. Introduction

In recent decades, teachers’ mental health has received increasing attention in the fields of educational psychology and occupational health, due to the high emotional, cognitive, and social demands inherent in teaching. Teaching, especially in secondary education, involves continuous interaction with students, managing disruptive behavior, dealing with institutional pressure, and constantly adapting to educational changes, placing this group among those most exposed to psychosocial risks (Li et al., 2020; Padmanabhanunni & Pretorius, 2023; Marić et al., 2020; Szigeti et al., 2017; Othman & Sivasubramaniam, 2019).

Anxiety and depression are among the most prevalent mental health problems in teachers and have a significant impact on both their well-being and the quality of the educational process (Othman & Sivasubramaniam, 2019). In the Spanish context, the study by Castillo Martínez et al. (2014) found that 16.4% of teachers exhibited symptoms of anxiety and 15% exhibited symptoms of depression, with both conditions co-occurring in 9.2% of cases. This reflects that psychological distress in teachers is not an isolated phenomenon, but rather a significant occupational health problem (Castillo Martínez et al., 2014). Internationally, evidence suggests even higher rates of impact. A recent study conducted in China found prevalences of 19.3% for anxiety and 34.7% for depression, as well as a 74.5% rate of burnout among teachers (He et al., 2025). Furthermore, international reviews have indicated that rates of anxiety and depression among teachers exceed those observed in the general population, placing this group as particularly vulnerable (Agyapong et al., 2022; Li et al., 2020).

Several individual, occupational, and contextual factors have been identified as contributors to these mental health problems. These include sociodemographic variables such as age and sex, as well as work-related factors such as workload, organizational demands, type of educational institution, and perceived social support (Marić et al., 2020; Li et al., 2020). In addition, the literature has also highlighted the importance of psychosocial factors in teaching, such as interaction with students, role ambiguity, and institutional pressure, as key elements in the development of psychological distress (He et al., 2025; Castillo Martínez et al., 2014).

Despite these advances, anxiety has traditionally been examined as a unitary construct, without considering its multidimensional nature. From a broader perspective, anxiety can be divided into different components, including cognitive anxiety (related to worries and anticipatory thoughts), physical anxiety (associated with physiological arousal responses), and social anxiety (linked to fear of negative evaluation in interpersonal contexts) (Clark & Beck, 2012; Eysenck et al., 2006). This distinction is especially relevant in the teaching field, where the demands of the educational environment can affect each of these dimensions differently (Mahan et al., 2010). Furthermore, the transdiagnostic approach has gained particular importance in the study of emotional disorders. Rather than conceptualizing anxiety and related conditions as independent diagnostic categories, this perspective proposes that different forms of psychological distress are maintained by common underlying mechanisms, including emotion regulation difficulties, excessive worry, rumination, anxiety sensitivity, and intolerance of uncertainty (Barlow et al., 2016; Harvey et al., 2004). By focusing on shared processes rather than specific diagnoses, the transdiagnostic framework facilitates a more comprehensive understanding of emotional maladjustment and its associated risk factors.

An increasing body of evidence supports the application of this perspective in educational settings. Recent systematic reviews have shown that transdiagnostic mechanisms are associated with a wide range of emotional difficulties and that school-based transdiagnostic interventions can effectively improve mental health and emotional wellbeing by targeting common psychological processes across disorders (Wang et al., 2024; Ghizzoni et al., 2025). In the teaching context, this perspective appears particularly relevant because occupational stressors frequently generate complex and overlapping emotional responses that cannot be fully understood through categorical diagnostic models alone (He et al., 2025; Castillo Martínez et al., 2014). Consequently, anxiety in teachers may be more accurately understood through the analysis of transdiagnostic vulnerability factors, which contribute simultaneously to different manifestations of emotional distress and may explain cognitive, physical, and social dimensions of anxiety.

Several transdiagnostic psychological variables have been consistently associated with anxiety and emotional maladjustment. Anxiety sensitivity refers to the tendency to fear anxiety-related sensations because they are perceived as having potentially harmful physical, cognitive, or social consequences. Individuals with high anxiety sensitivity are more likely to interpret normal physiological activation as threatening, increasing their vulnerability to anxiety symptoms. Intolerance of uncertainty, defined as the tendency to perceive uncertain situations as stressful, aversive, and difficult to tolerate, has been identified as one of the most robust transdiagnostic risk factors for anxiety and emotional distress (Morriss et al., 2023). Fear of negative evaluation, understood as apprehension regarding the possibility of being judged unfavorably by others, may be especially relevant in teaching due to the constant exposure to social and professional evaluation. Finally, procrastination has been linked to elevated levels of stress, anxiety, and emotional dysregulation, with recent evidence suggesting that procrastinatory behaviors contribute to psychological maladjustment in educational contexts (Ragusa et al., 2023). Despite the relevance of these variables, little research has examined their simultaneous contribution to different dimensions of anxiety among secondary school teachers.

Analyzing anxiety from a multidimensional perspective and integrating it with transdiagnostic variables allows for a more precise understanding of its underlying mechanisms and the factors that explain it. This approach not only contributes to the theoretical advancement of anxiety in teachers but also has important practical implications for the design of more effective preventive and therapeutic interventions, as it addresses core psychological processes that may underlie multiple manifestations of distress.

Based on the literature reviewed and the assumptions of the transdiagnostic framework, the following hypotheses were proposed:

**H1.**  *Higher levels of intolerance of uncertainty, fear of negative evaluation, and procrastination will be associated with higher levels of physical, cognitive, and social anxiety.*

**H2.**  *Transdiagnostic psychological variables will significantly predict the different dimensions of anxiety beyond the contribution of sociodemographic variables.*

**H3.**  *Intolerance of uncertainty and fear of negative evaluation will emerge as the strongest predictors of anxiety dimensions, reflecting their role as core transdiagnostic vulnerability factors.*

Therefore, the aim of this study is to analyze which psychological and sociodemographic factors predict the different dimensions of anxiety (physical, cognitive, and social) in secondary school teachers, considering previous evidence indicating the high prevalence of anxiety problems among teachers and the influence of personal and work-related variables, as well as shared psychological processes underlying its development.

## 2. Materials and Methods

### 2.1. Research Design and Participants

A quantitative, non-experimental, cross-sectional study was conducted to analyze the role of psychological and sociodemographic variables as predictors of the different dimensions of anxiety (physical, cognitive, and social) in secondary school teachers.

The study is framed within a correlational–explanatory approach with a variable-centered model, aimed at identifying relationships and predictive patterns between psychological and sociodemographic variables in relation to the different dimensions of anxiety. This type of design allows for the examination of associations between variables at a specific point in time, without experimental manipulation, making it suitable for studying psychological phenomena in natural contexts. Furthermore, this study is grounded in the transdiagnostic framework, which assumes that common psychological processes underlie a range of emotional disorders. This perspective conceptualizes anxiety not as a single construct but as a multidimensional phenomenon influenced by shared mechanisms involved in its development and maintenance.

The sample consisted of 444 Spanish secondary school teachers, 50% women (n = 222) and 50% men (n = 222), aged between 23 and 65 years old (M = 41.76; SD = 10.80). The majority were married or in a relationship (n = 293; 66%), and approximately half had children (n = 226; 50.9%). Regarding professional variables, teaching experience ranged from 0 to 39 years (M = 11.17; SD = 9.95), and 40.8% (n = 181) also held management positions. Detailed sample characteristics are presented in Table 1.

### 2.2. Procedure

Data collection was carried out from April to June 2025 using a self-administered online questionnaire distributed through the professional social network LinkedIn and targeted at Spanish secondary school teachers. The questionnaire was administered using Google Forms, and the estimated completion time was approximately 10–15 min.

Participation was voluntary and anonymous, and the confidentiality of responses was always guaranteed. Participants were informed beforehand of the study objectives and provided their informed consent. The ethical principles of research with human subjects and current data protection regulations were followed throughout the procedure. Also, the research plan was submitted to the Research Ethics Committee of the University of Burgos, and the required authorization was obtained under registration number 032.2/2021 (date of approval 6 February 2021). Participants were clearly informed of their right to withdraw from the study at any time during the research process without penalty or negative consequences. All procedures complied with current data protection regulations (GDPR and Organic Law 3/2018) and adhered to the ethical principles of the Declaration of Helsinki.

### 2.3. Measures

Relevant sociodemographic and professional variables were collected, including sex, age, marital status, having children, years of teaching experience, and performance of leadership roles. Dichotomous categorical variables were coded for regression analysis, with sex coded as 0 = male and 1 = female; having children as 0 = no and 1 = yes; marital status as 0 = single and 1 = in a relationship; and performance of leadership roles as 0 = no and 1 = yes. Age and years of teaching experience were treated as continuous variables.

Information was also obtained on anxiety sensitivity, which was the main variable of the study, as well as intolerance of uncertainty, procrastination, and fear of negative evaluation.

The Spanish version of the Anxiety Sensitivity Index-3 (ASI-3; Taylor et al., 2007; Sandín et al., 2007) was used to assess anxiety sensitivity. The scale consists of 18 items answered on a five-point Likert scale, where higher scores indicate greater sensitivity to anxiety. This instrument provides both a total score and specific scores on three distinct subscales:

- Sensitivity to physical anxiety assesses the fear of bodily sensations associated with physiological arousal (e.g., “It scares me when my heart beats rapidly”), based on the belief that they may have serious physical consequences (such as suffering a medical problem).
- Sensitivity to cognitive anxiety refers to the fear of the cognitive symptoms of anxiety, such as difficulty concentrating or a feeling of losing mental control (e.g., an item assessing fear of “going crazy” due to anxiety-related thoughts).
- Sensitivity to social anxiety measures the fear that visible manifestations of anxiety may be noticed by others and lead to negative evaluation or social rejection (e.g., an item assessing concern that others may notice visible signs of nervousness).

Validation studies of the Spanish version have shown adequate psychometric properties, highlighting high internal consistency for the total score, with Cronbach’s alpha coefficients greater than 0.90, as well as evidence of convergent validity with measures related to anxiety and psychological distress. In the present sample, the ASI-3 subscales showed good to excellent internal consistency. Cronbach’s alpha coefficients were 0.90 for sensitivity to physical anxiety, 0.90 for sensitivity to cognitive anxiety, and 0.85 for sensitivity to social anxiety. The Spanish version of the Intolerance of Uncertainty Scale—Short Form (IUS 12; Carleton et al., 2007; González Rodríguez et al., 2006) was used to assess intolerance of uncertainty. The scale consists of 12 items (e.g., “Unforeseen events upset me greatly”) answered on a five-point Likert scale, where higher values indicate greater levels of intolerance of uncertainty. The total score is obtained by summing the item scores. Validation studies in the Spanish population have demonstrated adequate psychometric properties of the instrument. Specifically, good internal consistency has been observed for the total score, with Cronbach’s alpha coefficients around 0.85, as well as adequate temporal stability in test–retest measures. In the present sample, the scale showed excellent internal consistency (Cronbach’s α = 0.88).

Fear of negative evaluation was assessed using the Brief Fear of Negative Evaluation Scale (BFNE; Leary, 1983), specifically its validated Spanish version (Gallego et al., 2007; Gallego, 2010). The scale consists of 12 items (e.g., an item assessing concerns about being judged negatively by others) answered using a five-point Likert scale. Four of the items are reverse-scored and were therefore recoded before calculating the total score, so higher scores reflect greater fear of negative evaluation. Validation studies in the Spanish population have shown adequate psychometric properties of the instrument. In particular, high levels of internal consistency have been reported, with Cronbach’s alpha coefficients greater than 0.88, as well as adequate indicators of composite reliability and evidence of convergent validity with other measures of social anxiety. In the present sample, the BFNE demonstrated excellent internal consistency (Cronbach’s α = 0.91).

Finally, the Spanish version of the Academic Procrastination Scale—Short Form (APS-S, Alegre Bravo et al., 2022) was used to assess academic procrastination. The APS-S consists of 5 items (e.g., an item assessing the tendency to delay tasks despite intending to complete them earlier), answered on a five-point Likert scale. Some items are worded in reverse, having been recoded prior to calculating the total score, so that higher scores indicate a greater tendency toward academic procrastination. The Spanish version of the APS-S has shown adequate psychometric properties, with excellent fit indices and high levels of reliability (Cronbach’s alpha coefficients greater than 0.87). In the present sample, the APS-S showed good internal consistency (Cronbach’s α = 0.80).

### 2.4. Analysis

Data analysis was performed using IBM SPSS Statistics version 29.0. First, descriptive statistics (mean and standard deviation) were calculated for the main study variables. No missing data were observed in the final dataset, as only fully completed questionnaires were included in the analyses. Subsequently, bivariate relationships between variables were analyzed using Pearson correlation coefficients, as these were continuous quantitative variables, following recommendations for continuous quantitative data (Field, 2018). The aim was to explore preliminary associations between the transdiagnostic psychological variables (intolerance of uncertainty, fear of negative evaluation, and procrastination) and the different dimensions of anxiety (physical, cognitive, and social).

To analyze the predictive capacity of the independent variables, hierarchical multiple regression analyses were conducted for each anxiety dimension (physical anxiety, cognitive anxiety, and social anxiety), as well as for total anxiety, following established recommendations for predictive analyses (Tabachnick & Fidell, 2019). Variables were entered in three sequential blocks. In Step 1, sociodemographic variables (sex, age, having children, and marital status) were entered. In Step 2, work-related variables (years of teaching experience and holding a leadership role) were added. Finally, in Step 3, the transdiagnostic psychological variables (intolerance of uncertainty, fear of negative evaluation, and procrastination) were entered. This hierarchical approach made it possible to examine the incremental contribution of psychological variables beyond the variance explained by sociodemographic and work-related factors.

For each model, unstandardized coefficients (B), standardized coefficients (β), t values, significance levels (p), coefficient of determination (R^2^), adjusted coefficient of determination (adjusted R^2^), and changes in explained variance (ΔR^2^) were examined. The assumptions of the regression model were also verified, including the normality of the residuals, homoscedasticity, independence of errors (using the Durbin–Watson statistic), and the absence of multicollinearity among the predictors, assessed through tolerance values and the variance inflation factor (VIF). VIF values below 5 were considered indicative of the absence of problematic collinearity (Hair et al., 2019).

A level of statistical significance of *p* < 0.05 was established in all analyses.

## 3. Results

Pearson correlations between the main variables of the study were analyzed (Table 2).

The psychological variables showed significant positive correlations with each other. Intolerance of uncertainty and fear of negative evaluation showed moderate-to-high associations with the different dimensions of anxiety. Likewise, procrastination was positively correlated with cognitive and social anxiety, although to a lesser extent.

Hierarchical multiple regression analyses were conducted for each anxiety dimension (Table 3). Demographic variables (sex, age, children, and marital status) were entered in Step 1, work-related variables (teaching experience and leadership role) in Step 2, and psychological variables (intolerance of uncertainty, fear of negative evaluation, and procrastination) in Step 3.

For physical anxiety, demographic variables explained 7.4% of the variance (R^2^ = 0.074, *p* < 0.001). The inclusion of work-related variables did not significantly improve the model (ΔR^2^ = 0.011, *p* = 0.069). However, the addition of psychological variables significantly increased the explained variance by 17.2% (ΔR^2^ = 0.172, *p* < 0.001). The final model explained 25.7% of the variance (R^2^ = 0.257, adjusted R^2^ = 0.242). Intolerance of uncertainty emerged as the strongest predictor (β = 0.35, *p* < 0.001), followed by fear of negative evaluation (β = 0.16, *p* < 0.001) and sex (β = 0.16, *p* < 0.001).

For cognitive anxiety, demographic variables explained 6.9% of the variance (R^2^ = 0.069, *p* < 0.001). Work-related variables did not significantly increase the explained variance (ΔR^2^ = 0.008, *p* = 0.144). The inclusion of psychological variables significantly improved the model, accounting for an additional 23.4% of the variance (ΔR^2^ = 0.234, *p* < 0.001). The final model explained 31.2% of the variance (R^2^ = 0.312, adjusted R^2^ = 0.298). Intolerance of uncertainty was the strongest predictor (β = 0.38, *p* < 0.001), followed by fear of negative evaluation (β = 0.18, *p* < 0.001), teaching experience (β = −0.14, *p* = 0.037), procrastination (β = 0.12, *p* = 0.007), and sex (β = 0.11, *p* = 0.011).

For social anxiety, demographic variables explained 8.1% of the variance (R^2^ = 0.081, *p* < 0.001). The inclusion of work-related variables did not significantly improve the model (ΔR^2^ = 0.001, *p* = 0.721). Psychological variables significantly increased the explained variance by 38.4% (ΔR^2^ = 0.384, *p* < 0.001). The final model accounted for 46.6% of the variance (R^2^ = 0.466, adjusted R^2^ = 0.455). Fear of negative evaluation emerged as the strongest predictor (β = 0.39, *p* < 0.001), followed by intolerance of uncertainty (β = 0.35, *p* < 0.001). Procrastination (β = 0.11, *p* = 0.005) and sex (β = 0.11, *p* = 0.004) were also significant predictors.

Finally, total anxiety was also analyzed using hierarchical multiple regression (Table 4). Demographic variables entered in Step 1 explained 9.5% of the variance (R^2^ = 0.095, *p* < 0.001). The inclusion of work-related variables in Step 2 did not significantly improve the model (ΔR^2^ = 0.008, *p* = 0.162). In Step 3, psychological variables significantly increased the explained variance by 32.6% (ΔR^2^ = 0.326, *p* < 0.001). The final model explained 42.9% of the variance in total anxiety (R^2^ = 0.429, adjusted R^2^ = 0.417). Intolerance of uncertainty emerged as the strongest predictor (β = 0.41, *p* < 0.001), followed by fear of negative evaluation (β = 0.28, *p* < 0.001), sex (β = 0.14, *p* < 0.001), and procrastination (β = 0.10, *p* = 0.015).

Overall, the hierarchical regression analyses revealed that psychological variables contributed substantially more to the explanation of anxiety dimensions than demographic or work-related factors. Across all models, the inclusion of transdiagnostic psychological variables produced the largest increases in explained variance, ranging from 17.2% for physical anxiety to 38.4% for social anxiety. Intolerance of uncertainty emerged as the most consistent predictor across all anxiety dimensions and total anxiety, whereas fear of negative evaluation showed its strongest effect on social anxiety. Procrastination was specifically associated with cognitive, social, and total anxiety. These findings support the relevance of transdiagnostic psychological processes in understanding anxiety among secondary school teachers.

## 4. Discussion

### 4.1. The Multidimensional Nature of Anxiety and the Role of Transdiagnostic Processes

This study aimed to analyze the role of transdiagnostic psychological and sociodemographic variables as predictors of different dimensions of anxiety in secondary school teachers. The results provide solid evidence that teacher anxiety is not a unitary construct but rather presents a multidimensional structure with differentiated prediction patterns depending on each dimension analyzed (physical, cognitive, and social). This finding is consistent with contemporary approaches that conceptualize anxiety as a heterogeneous phenomenon, composed of multiple partially independent response systems. Furthermore, the findings support the usefulness of the transdiagnostic framework for understanding teacher anxiety, as common psychological processes were associated with multiple anxiety dimensions despite their distinct manifestations. The hierarchical regression analyses further revealed that transdiagnostic psychological variables accounted for substantially more variance in anxiety dimensions than sociodemographic and work-related factors, highlighting their central role in understanding teachers’ emotional distress.

### 4.2. The Contribution of Transdiagnostic Psychological Variables to Teacher Anxiety

First, intolerance of uncertainty emerged as the most consistent and robust predictor across all dimensions of anxiety, showing the greatest explanatory power for total anxiety as well as cognitive and social anxiety. This result supports the central role of this variable as a transdiagnostic mechanism involved in different forms of anxiety (Carleton, 2016; Dugas et al., 1998). The finding is also consistent with more recent evidence identifying intolerance of uncertainty as one of the most robust transdiagnostic vulnerability factors underlying emotional disorders and psychological distress (Morriss et al., 2023). From a theoretical perspective, intolerance of uncertainty is related to the tendency to perceive ambiguous situations as threatening, generating cognitive hypervigilance and negative anticipation. In the teaching context, characterized by changing demands, ambiguity in decision-making, and constant evaluation, this cognitive pattern can intensify the perception of psychological burden and contribute to the development of anxious responses, in line with evidence pointing to the role of job demands in teachers’ psychological distress (Castillo Martínez et al., 2014; He et al., 2025).

Furthermore, the strong association observed between intolerance of uncertainty and cognitive anxiety supports the idea that this dimension of anxiety is especially linked to processes of worry, rumination, and difficulties in thought control. This finding is consistent with previous research that has indicated that uncertainty is a key trigger for sustained worry processes (Dugas et al., 1998), which in the educational field can translate into anticipation of errors, doubts about professional performance or excessive concern about student performance. Taken together, these findings reinforce recent transdiagnostic perspectives suggesting that intolerance of uncertainty acts as a common mechanism linking diverse forms of anxiety and emotional maladjustment (Morriss et al., 2023).

Secondly, fear of negative evaluation emerged as the most significant predictor of social anxiety, exhibiting the strongest effect in this dimension. This result is conceptually consistent, given that the construct of social anxiety is defined precisely by concern about the negative judgment of others (Leary, 1983). In the teaching context, this finding is particularly relevant, as educational practice involves constant exposure to evaluative situations, both formal and informal, which can trigger concerns related to professional competence, student acceptance, or evaluation by other educational stakeholders—aspects widely described as sources of stress for teachers (Castillo Martínez et al., 2014). Furthermore, the relatively high correlation between fear of negative evaluation and social anxiety observed in previous analyses highlights the internal consistency of the model. In line with the previous literature, these results suggest that social anxiety in work contexts is closely linked to the perception of external evaluation and the fear of criticism (McLean & Anderson, 2009), which is especially relevant in professions such as teaching, where public exposure and social interaction are constant. From a transdiagnostic perspective, this finding suggests that concerns about social evaluation may represent a shared vulnerability process that contributes not only to social anxiety but also to broader emotional difficulties.

On the other hand, procrastination showed a differential pattern, being significant in predicting cognitive and social anxiety, but not physical anxiety. This result makes a relevant contribution, as it suggests that procrastination is primarily associated with cognitive and behavioral avoidance processes, rather than physiological arousal. According to Steel’s model (2007), procrastination can be understood as a short-term emotional regulation strategy, whereby people avoid tasks that generate discomfort, which in the long-term increases anxiety. From a transdiagnostic perspective, this finding reinforces the idea that avoidance behaviors constitute a central mechanism shared in various emotional problems. Recent evidence has similarly highlighted the relationship between procrastination, emotional dysregulation, stress, and anxiety, supporting its relevance as a transdiagnostic process in educational contexts (Ragusa et al., 2023).

### 4.3. Sociodemographic and Occupational Factors Associated with Anxiety

Concerning sociodemographic variables, sex showed a significant effect across all dimensions analyzed, revealing higher levels of anxiety in women. This result is consistent with previous evidence that identifies women as a more vulnerable group in relation to anxiety disorders and teacher burnout. Explanations for this pattern have been varied, including biological, social, and cultural factors, as well as differences in emotional socialization and in the perception of job demands (He et al., 2025).

Meanwhile, teaching experience showed a protective effect on cognitive anxiety, suggesting that the accumulation of professional experience can contribute to the development of more effective coping strategies. This result is consistent with previous research indicating that more experienced teachers tend to exhibit better psychological adjustment and a lower perception of threat in the face of job demands. In this sense, experience could act as a modulating factor that reduces perceived uncertainty and increases confidence in one’s own classroom management abilities (Castillo Martínez et al., 2014).

In contrast, variables such as age, having children, marital status, or holding leadership roles did not show significant effects in the analyzed models. Moreover, the hierarchical regression analyses showed that sociodemographic and work-related variables contributed only modestly to the explanation of anxiety, whereas the incorporation of psychological variables resulted in substantial increases in explained variance across all models. This finding reinforces the usefulness of the transdiagnostic approach, highlighting the central role of emotional and cognitive processes versus more general structural variables (Steel, 2007).

### 4.4. Practical Implications

Overall, the results of this study advance the understanding of teacher anxiety from a transdiagnostic perspective, demonstrating that different dimensions of anxiety are associated with distinct, yet partially shared, psychological mechanisms. Notably, the hierarchical regression analyses revealed that transdiagnostic psychological variables accounted for the largest increases in explained variance across all anxiety dimensions, particularly for social anxiety, whereas demographic and work-related factors contributed comparatively little. This finding further supports the importance of transdiagnostic processes in explaining teacher anxiety beyond demographic and occupational characteristics. This multidimensional approach is particularly relevant for the design of interventions, as it suggests that not all forms of anxiety should be addressed in the same way, but rather require interventions tailored to the specific underlying processes. From an applied perspective, the results underscore the need to develop intervention programs that include components aimed at improving uncertainty tolerance, cognitive restructuring, social evaluation management, and reducing avoidance behaviors such as procrastination. These types of interventions, aligned with transdiagnostic models, can be particularly effective by addressing core processes common to different manifestations of anxiety.

More specifically, the findings have important practical implications for teacher training, occupational health, and psychological intervention. From a preventive perspective, educational institutions could implement programs aimed at strengthening teachers’ ability to cope with uncertainty, manage evaluation-related concerns, and develop adaptive emotional regulation strategies. In addition, school-based interventions derived from transdiagnostic approaches have shown promising results in promoting emotional wellbeing by targeting common psychological mechanisms rather than disorder-specific symptoms (Wang et al., 2024; Ghizzoni et al., 2025). Early identification of teachers with elevated levels of intolerance of uncertainty, fear of negative evaluation, or procrastination may facilitate the implementation of prevention strategies capable of reducing anxiety symptoms before they become clinically significant. Furthermore, these findings suggest that particular attention should be given to female teachers and less experienced professionals, who may represent groups at increased risk for anxiety-related difficulties.

### 4.5. Limitations and Future Research

However, this study has some limitations that should be considered. First, the cross-sectional design prevents establishing causal relationships between the variables analyzed, so future research should adopt longitudinal designs that allow for examining the evolution of these processes over time. Second, the use of self-report measures may introduce biases associated with social desirability or the participants’ subjective perceptions. In addition, because all variables were assessed using self-report measures, the possibility of common method bias cannot be completely ruled out. Shared measurement characteristics may have inflated some of the observed associations between variables. Future research would benefit from incorporating multi-method assessment strategies, including behavioral indicators, observational measures, or reports from external informants, to reduce the potential impact of common method variance. It would also be advisable to include additional measures to enrich the assessment of these constructs. Finally, future research could explore the role of organizational variables such as school climate, institutional support, and workload—factors that have been previously identified as relevant to teachers’ mental health.

## 5. Conclusions

The present study contributes to the understanding of anxiety among secondary school teachers from a multidimensional and transdiagnostic perspective. The findings indicate that intolerance of uncertainty, fear of negative evaluation, and procrastination are significant predictors of different anxiety dimensions, whereas sociodemographic variables show a comparatively smaller contribution. Among the variables examined, intolerance of uncertainty emerged as the most consistent predictor across anxiety dimensions, while fear of negative evaluation was particularly relevant for social anxiety. These results support the relevance of transdiagnostic mechanisms in understanding teacher anxiety and indicate that psychological variables explain a substantially greater proportion of variance in anxiety than sociodemographic or work-related factors. Consequently, interventions targeting common psychological processes may be especially effective in promoting teacher wellbeing. Future research should employ longitudinal designs and incorporate contextual and organizational variables to further clarify the mechanisms underlying anxiety in educational professionals.

## Figures and Tables

**Table 1:** Sociodemographic characteristics of the sample.

Variable	n	%	M	SD	Range
**Gender**					
Male	222	50.0			
Female	222	50.0			
**Marital status**					
Single	151	44.0			
Married/in a relationship	293	66.0			
**Children**					
No children	218	49.1			
With children	226	50.9			
**Professional role**					
Teacher	263	59.2			
School management/coordination	181	40.8			
**Age (years)**			41.76	10.80	23–65
**Years of teaching experience**			11.17	9.95	0–39

Note. n = frequency; M = mean; SD = standard deviation.

**Table 2:** Pearson correlations between psychological variables.

Variable	M	SD	Min	Max	Skewness	Kurtosis	ASI-3 P	ASI-3 C	ASI-3 S	IUS-12	BFNE	APS-S
ASI-3 P	11.85	5.64	6	29	0.97	0.28	—					
ASI-3 C	10.80	5.32	6	30	1.36	1.50	0.75 ***	—				
ASI-3 S	14.15	5.68	6	30	0.60	−0.37	0.59 ***	0.62 ***	—			
IUS-12	32.48	8.68	12	60	0.24	−0.07	0.44 ***	0.49 ***	0.53 ***	—		
BFNE	33.37	10.71	8	60	0.22	−0.23	0.33 ***	0.39 ***	0.58 ***	0.39 ***	—	
APS-S	11.67	5.50	4	25	0.35	−0.77	0.13 **	0.23 ***	0.27 ***	0.16 ***	0.30 ***	—

Note. ASI-3 P = Sensitivity to physical anxiety; ASI-3 C = Sensitivity to cognitive anxiety; ASI-3 S = Sensitivity to social anxiety; IUS-12 = Intolerance of Uncertainty Scale—Short Form; BFNE = Brief Fear of Negative Evaluation Scale; APS-S = Academic Procrastination Scale—Short Form. ** *p* < 0.01, *** *p* < 0.001.

**Table 3:** Hierarchical regression analyses predicting anxiety dimensions (final models).

Predictor	Physical Anxiety B (β)	t	*p*	Cognitive Anxiety B (β)	t	*p*	Social Anxiety B (β)	t	*p*
Sex	1.77 (0.16)	3.67	<0.001	1.12 (0.11)	2.58	0.011	1.21 (0.11)	2.94	0.004
Age	0.03 (0.06)	0.86	0.392	0.03 (0.07)	0.95	0.345	0.02 (0.03)	0.52	0.604
Children	0.24 (0.02)	0.36	0.717	0.01 (0.00)	0.01	0.993	−0.24 (−0.02)	−0.43	0.667
Marital status	−0.77 (−0.07)	−1.39	0.164	−0.43 (−0.04)	−0.85	0.398	−0.06 (−0.01)	−0.13	0.894
Teaching experience	−0.06 (−0.10)	−1.37	0.173	−0.08 (−0.14)	−2.06	0.037	−0.03 (−0.06)	−0.95	0.345
Leadership role	−0.35 (−0.03)	−0.66	0.510	0.26 (0.02)	0.54	0.589	0.42 (0.036)	0.91	0.361
IUS-12	0.22 (0.35)	7.41	<0.001	0.23 (0.38)	8.46	<0.001	0.23 (0.35)	8.92	<0.001
APS-S	0.03 (0.03)	0.64	0.522	0.11 (0.12)	2.73	0.007	0.11 (0.11)	2.81	0.005
BFNE	0.08 (0.16)	3.33	<0.001	0.09 (0.18)	3.82	<0.001	0.21 (0.39)	9.68	<0.001

Note. B = unstandardized regression coefficient; β = standardized regression coefficient. IUS-12 = Intolerance of Uncertainty Scale–Short Form; BFNE = Brief Fear of Negative Evaluation Scale; APS-S = Academic Procrastination Scale–Short Form. Physical anxiety: R^2^ = 0.257; Adjusted R^2^ = 0.242; ΔR^2^ = 0.172. Cognitive anxiety: R^2^ = 0.312; Adjusted R^2^ = 0.298; ΔR^2^ = 0.234. Social anxiety: R^2^ = 0.466; Adjusted R^2^ = 0.455; ΔR^2^ = 0.384.

**Table 4:** Hierarchical regression analysis predicting total anxiety (final model).

Predictor	B	β	t	*p*
Sex	4.10	0.14	3.74	<0.001
Age	0.08	0.06	0.95	0.342
Children	0.00	0.00	0.00	0.999
Marital status	−1.26	−0.04	−1.00	0.317
Teaching experience	−0.16	−0.11	−1.80	0.073
Leadership role	0.33	0.01	0.27	0.787
IUS-12	0.69	0.41	10.00	<0.001
APS-S	0.25	0.10	2.43	0.015
BFNE	0.38	0.28	6.64	<0.001

Note. B = unstandardized regression coefficient; β = standardized regression coefficient. IUS-12 = Intolerance of Uncertainty Scale–Short Form; BFNE = Brief Fear of Negative Evaluation Scale; APS-S = Academic Procrastination Scale–Short Form. R^2^ = 0.429; Adjusted R^2^ = 0.417; ΔR^2^ Step 3 = 0.326. Dependent variable: Total anxiety.

## Data Availability

The data presented in this study are available on request from the corresponding author due to privacy and ethical considerations.
